# Functional Design Analysis of Two Current Extended-Depth-of-Focus Intraocular Lenses

**DOI:** 10.1167/tvst.13.8.33

**Published:** 2024-08-21

**Authors:** Damian Mendroch, Uwe Oberheide, Stefan Altmeyer

**Affiliations:** 1 Institute for Applied Optics and Electronics, Cologne University of Applied Sciences, Cologne, North Rhine-Westphalia, Germany

**Keywords:** intraocular lens, cataract, raytracing, simulation, lens metrology, extended depth of focus, mathematical modelling

## Abstract

**Purpose:**

To evaluate the differences between two extended depth-of-focus intraocular lenses, the Alcon IQ Vivity and the Bausch & Lomb LuxSmart and to compare them with a simple monofocal lens, the Alcon IQ, using a simulation-based approach.

**Methods:**

A mathematical lens model was created for each lens type based on a measured surface geometry. The lens model was then used in a raytracer to calculate a refractive power map of the lens and a ray propagation image for the focal zone.

**Results:**

The simulations confirm the enhanced depth of focus of these two lenses. There are apparent differences between the models. For the Vivity, more light is directed into the far focus in low light conditions, whereas the LuxSmart behaves more pupil independent and prioritizes intermediate vision.

**Conclusions:**

The simulation-based approach was effective in evaluating and comparing the design aspects of these lenses. It can be positioned as a valuable third tool for lens characterization, complementing in vivo studies and in vitro measurements.

**Translational Relevance:**

With this approach not only focusing on the resulting optical performance, but the underlying functional mechanisms, it paves the way forward for a better adaptation to the individual needs and preferences of patients.

## Introduction

Extended depth of focus intraocular lenses (EDoF IOL) provide a third, newer option for cataract surgery, along with the monofocal and multifocal lens technologies. Unlike monofocal lenses, which only provide clear vision at far distances, these lenses also offer acceptable vision at intermediate distances. However, high quality of vision at near distances, like done with a multifocal IOL, is not achieved. By contrast, the elongated focus of an EDoF IOL, which typically provides a focal range of 1.0 to 1.5 diopter (D or dpt), decreases photic phenomena like halos and glares.^1^^,^^2^ Trifocal and especially bifocal lenses are more susceptible to these disturbing effects because of their distinct focal positions and the resulting less smooth defocus curve. These lenses are often diffractive in nature and the light that is lost in unusable higher diffraction orders further lowers the contrast. The advantages of the EDoF IOL also include increased contrast sensitivity and, for some models, greater tolerance to decentration than multifocal IOLs.^1^^,^^3^ A greater tolerance to refractive shift, power mismatch and residual astigmatism is also expected.^4^^–^^6^ Many patients are not suitable candidates for multifocal lenses due to pathological manifestations like epiretinal membrane, age-related macular degeneration, corneal conditions, or because of concerns about decreased contrast sensitivity and positive dysphotopsia.^7^^,^^8^ For some of these patients, EDoF IOLs may still offer a chance to decrease their dependence on glasses.

Different means of comparison for lens models and types would lead to a better understanding and an easier selection process, benefiting both ophthalmologists and patients. Researchers typically characterize these lenses either in in vivo studies or in in vitro measurements. In the latter case, the standard ISO 11979-2 specifies measurements, setup, and evaluation criteria.^9^ However, such a process is limited by issues that arise directly from its physical nature, including the need for equipment and objects, precise adjustment, and lack of flexibility in changing the conditions of the setup. A simulation-based approach, in contrast, would simplify the procedure, decrease the effort considerably, make the results reproducible, and enable further possibilities that are difficult or impossible to realize in the measurement setup. For example, incorporating the patient's individual eye geometry, applying a defined lens tilt or using an arbitrary light spectrum are much easier to realize digitally.

The purpose of this work was to evaluate the differences between two current EDoF lenses, the Alcon IQ Vivity, and the Bausch & Lomb LuxSmart and to compare them with a simple monofocal lens, the Alcon IQ. This comparison is made by creating a mathematical lens model and using a software-based simulation. Along the way, a method to derive optical properties from the surface geometry and the optical power profile will be presented, that can also be valuable for research on similar lenses.

Last, this research work builds on top of earlier approaches and results from other works. Miret et al.^10^ deduced optical properties from the measured surface geometry of various types of IOLs. The performance of various aspheric IOL models has been assessed by Barbero et al.^11^ by simulation. Tognetto et al.^12^^,^^13^ measured the surface profiles of different EDoF lenses by using profilometry.

## Methods

### Lenses

The lenses under investigation include two purely refractive EDoF IOLs with different mechanisms for focus extension, as well as a standard monofocal lens as reference. For ease of comparison, all lenses share the same refractive power of 20 D. The commonly used Fraunhofer FdC spectral lines (486.13 nm, 587.56 nm, and 656.28 nm) are assumed for the subsequent indices of refraction and Abbe numbers, although this is not mentioned specifically in the references.

### Alcon IQ

The AcrySof IQ (Alcon Laboratories Inc., Fort Worth, TX), model number SN60WF, is a monofocal, foldable, single piece lens with a biconvex design.^14^ The lens is made of a hydrophobic acrylic material with a refractive index of n = 1.5542 and an Abbe number of V = 37.^15^^,^^16^ The posterior side is aspheric and adds −0.20 µm of negative spherical aberration.^14^^,^^16^ An associated patent describes the mathematical surface formulation, as well as the lens thickness, and will prove useful for simulation.^17^^,^^18^

### Alcon IQ Vivity

The AcrySof IQ Vivity (Alcon Laboratories Inc.), model number DFT015, is an EDoF lens based on the Alcon IQ platform.^19^ It is also a foldable, single piece, hydrophobic, and biconvex lens and made of the same acrylic material as the IQ model.^19^^–^^21^ Although the posterior side is spherical, the anterior side is aspheric and features the nondiffractive X-Wave Technology, a wavefront shaping design that is discussed in detail in the corresponding patent application.^17^^,^^20^^,^^22^ As per the manufacturer, the focal range is given as 1.5 D and the spherical aberration correction is claimed as −0.20 µm.^23^

### Bausch & Lomb LuxSmart

Last, the LuxSmart (Bausch & Lomb Inc., Bridgewater Township, NJ), model number YSMART, also includes an EDoF lens design. The single piece lens consists of an acrylic hydrophobic material with a refractive index of n = 1.540 and an Abbe number of V = 43.^23^^,^^24^

The lens is composed of three zones. Its inner part is a focus center with a 2-mm diameter, combining spherical aberration of the fourth and sixth order with opposite signs to achieve a higher depth of focus. The lens periphery is monofocal and aspheric, while the zone in between is a patented, smooth transition zone between the former two.^25^ The range of useful vision is specified as 1.5 D, and the lens is aberration neutral; therefore, it does not compensate any positive spherical aberration of the cornea.^23^ Rocha et al.^26^ demonstrated previously an increased depth of focus by an addition of spherical aberration of the fourth order, whereas other works showed an even stronger effect for a combination with the sixth order of the opposite sign.^27^^,^^28^

Although there are no statements on the exact patent for the transition zone, we assume it is the same as that for the Synthesis+ IOL (Cutting Edge S.A.S., Montpellier, France).^29^^,^^30^ This EDoF model has a similar surface topology and a likewise patented transition zone as the Bausch & Lomb lens. To our knowledge, there are no other comparable patents and, because Cutting Edge manufactures the LuxSmart model, a cooperation in knowledge exchange also seems likely.^31^

### Lens Fitting

Before proceeding to the analysis and simulation steps, mathematical lens models must be constructed. A confocal microscope measured the surface geometry of the lens, resulting in a three-dimensional dataset. This dataset is then centered, tilt corrected, and disassembled into radial profiles. A mean profile is obtained by averaging these radial profiles and is then decomposed into a conic section, polynomial and, if any, a remaining differential part. More details are available in our previous research work, which applies the same approach for multifocal IOLs.^32^

### Simulation

Virtual retinal imaging is done with the help of a self-written simulator. The simulator developed is a sequential raytracer that models geometric optics using a Monte Carlo approach.

The raytracer creates rays randomly according to the intensity and geometry distribution of the object. The wavelength and initial ray direction are assigned depending on the desired spectral and geometrical lightning distribution. Surface intersection calculation is done analytically for surfaces of types plane, sphere, and conic section and numerically for aspheric or arbitrary surface functions. After the calculation of the ray–surface intersection the new ray direction is calculated with the three-dimensional version of Snell's law. Besides refractive surfaces, it is also possible to define absorptive surfaces that act as apertures.

Lens materials or ambient media are implemented as either dispersive or wavelength independent. For dispersive media, the refractive index is modelled by an index value *n* at a reference wavelength and the Abbe number *V*. With these two parameters and known reference wavelengths a model of the following form is calculated:

$$\begin{array}{c} n\left(\lambda \right)=A+\frac{B}{\;\lambda^{2}-\;\lambda_{0}^{2}}, \end{array}$$

where the value of $\lambda_{0}^{2}=0.014$ µm^2^ is a compromise between the Cauchy model ($\lambda_{0}^{2}=0.0$ µm^2^) and the Herzberger formula ($\lambda_{0}^{2}=0.028$ µm^2^).^33^ Although the behavior of materials with the same *n* and *V* can differ slightly, especially outside the region for which these properties are defined, a similar behavior inside the visible range is expected.

### Power Profile

Optical power profiles are a valuable tool to characterize the refractive behavior over the entrance pupil of the lens, assess power zones and estimate spherical aberrations. Research work analyzing multifocal contact lenses often provides this kind of plot.^34^^–^^36^ Del Águila-Carrasco et al.^37^ went as far as presenting a method to estimate wavefront errors from a measured power map. Here, we take a similar approach.

Iskander et al.^38^ present a formulation for power deviations due to wavefront errors relative to a planar wave, whereas Wyant and Creath^39^ show a similar formulation for errors relative to spherical waves. Iskander et al.^40^ demonstrate in another publication that, for the eye geometry and small wavefront errors, both approaches are equivalent. For simplicity, we prefer the former. The power deviation Δ*D* at radial position *r* for the radially symmetric wavefront error *W*(*r*) is defined as^38^:

$$\begin{array}{c} \Delta D\left(r\right)=\frac{1}{r}\;\frac{d\;W\;\left(r\right)}{d\;r} \end{array}$$

In the case of fourth- and sixth-order Seidel aberrations *W*_4,6_(*r*) = *ar*^6^ + *br*^4^ the equation results in:

$$\begin{array}{c} \Delta D_{4,6}\left(r\right)=6ar^{4}+4br^{2} \end{array}$$

**Figure 1.**
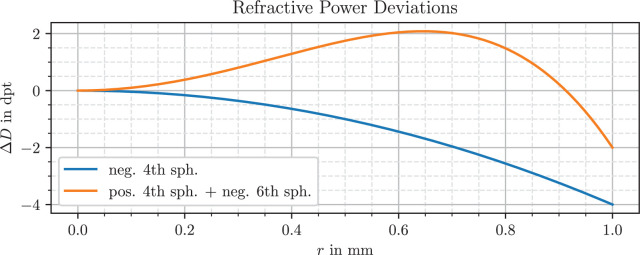
Exemplary Δ*D*_4,6_ curves. The blue curve shows pure fourth-order spherical aberration with *a* = 0.000 mm^−5^_,_ *b* = −0.001 mm^−3^ and the orange one a combination of both types with *a* = −0.002 mm^−5^_,_ *b* = 0.0025 mm^−3^.

A lens without aberrations has a constant power across the entrance pupil, and a lens with only fourth-order spherical aberration has either a radially increasing power for *b* > 0 or a decreasing one for *b* < 0. Downward-facing parabolas are a known profile shape for IOLs with negative spherical aberration, from multiple sources.^41^^,^^42^ An example for a lens combining fourth- and sixth-order spherical aberration is the Zeiss CT Lucia (Carl Zeiss Meditec, Jena, Germany), which incorporates zones with varying spherical aberrations.^41^ Figure 1 shows two exemplary profiles of aberration combinations.

## Results

### Base Shape

**Figure 2.**
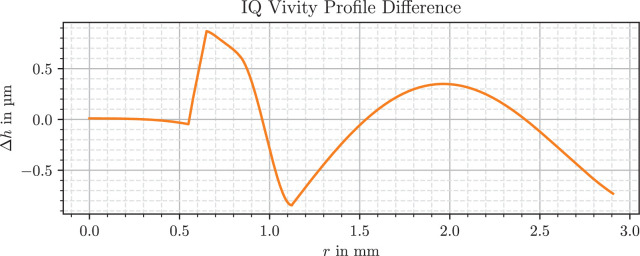
Filtered difference to best-fit conic section for the anterior side of the IQ Vivity lens.

**Figure 3.**
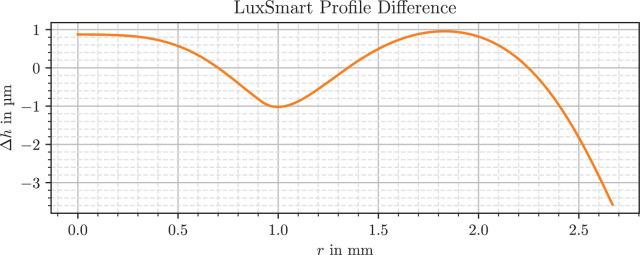
Filtered difference to best-fit conic section for the anterior side of the LuxSmart lens.

The modelling approach from 2.2 leads to a mathematical formulation for both lens shapes. Both IOLs are clinically relevant and state-of-the-art models that are protected by intellectual property. Only parts of their model are presented here. Figures 2 and 3 present the deviations of the profile to the best-fit conic section of the anterior side of the lens, which is the surface responsible for enhanced focus. The basic shape has been removed, which as a conic section can be either a sphere, parabola, hyperbola, or an ellipse, so only polynomial components and higher-order structures remain.

The profile of the Vivity lens has a flat center up to 0.55 mm and a larger curved periphery beyond 1.10 mm. The wavefront-shaping design between these two zones consists of three apparently linear segments that create an elevation of almost 1 µm. As demonstrated in the referenced video, these ring structures are also already detectable with the help of appropriate lighting.^43^

The LuxSmart difference profile consists of two differently curved sections divided at *r* = 1 mm, and the inner zone is smaller than the outer periphery. The transition between them is constructed smoothly. A special edge design limits the available active region of the lens from the initial optical radius of 3.0 mm to less than 2.7 mm. With height differences of more than 4 µm, the peak-to-peak shape difference is larger than that of the Vivity with 1.7 µm.

Interestingly, both models divide the inner and outer part of the lens at around *r* = 1 mm, equivalent to a pupil size of *P* = 2 mm. And in both cases, shape deviations in the low micron range seem to be sufficient for the creation of the enhanced depth of focus.

### Power Profile

The ISO 11979-2 defines the measurement setup and the mathematical formalism for measuring the refractive power of IOLs.^9^ A measurement object is set to a distance of infinity on an optical bench and image by the IOL. A downstream microscope is used to determine the focal point position for a specific cut-off frequency of the modulation transfer function (MTF). From this, the lens power is calculated according to equations A4 to A8 of the standard, while taking into account the position of the second principal plane and a defocus correction term resulting from the longitudinal spherical aberration.

**Figure 4.**
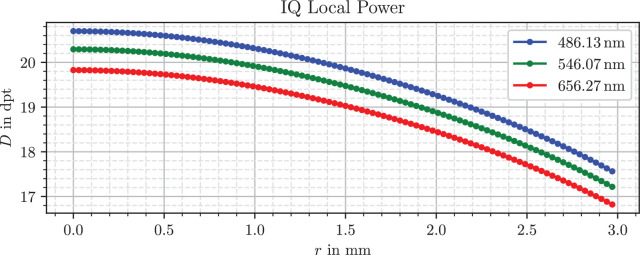
Refractive power profile of the Alcon IQ.

**Figure 5.**
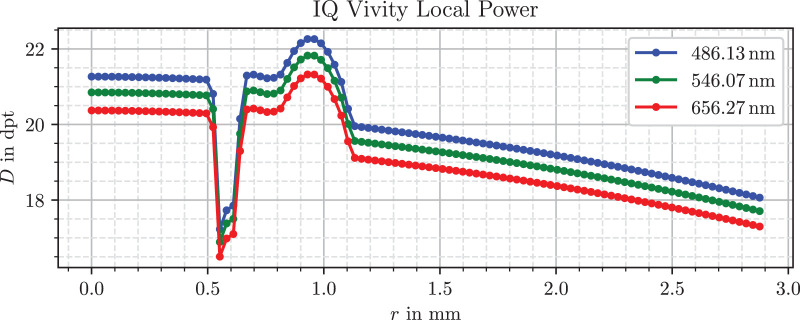
Refractive power profile of the Alcon IQ Vivity lens.

**Figure 6.**
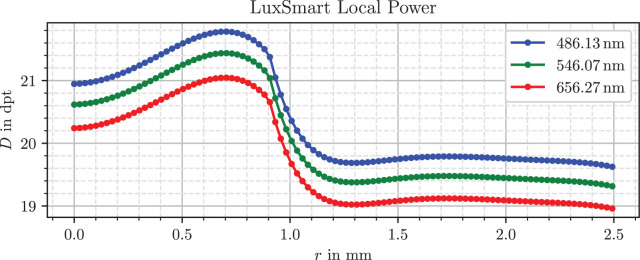
Refractive power profile of the Bausch & Lomb LuxSmart.

For our application, we strongly simplify the modelling effort by stationing the lens directly inside the vitreous medium of *n_v_* = 1.336 and irradiating the lens with light parallel to the optical axis, which can be generated easily in the simulation. To measure local refractive powers, a ring source illuminates only a small radial section of the lens, for which the focal length can be determined easily and clearly. The calculations are still in line with the standard; however, the defocus correction term was neglected, because we do not calculate an equivalent total refractive power, but a local one. Because all lenses are approximately symmetrically biconvex, a simplified ratio of front and overall lens power *D_f_/D* ≈ 0.5 was assumed for the principal plane equation A4 of the standard. For each combination of lens model, ring radius and wavelength the optical power was determined. Figures 4, 5, and 6 show the resulting plots.

The monofocal Alcon IQ, shown in Figure 4, has a central optical power of slightly greater than 20 D for the green wavelength and a downward-facing parabola curve shape, which indicates negative spherical aberration. At the edge of the lens the power has decreased down to 17.2 D, an absolute difference of approximately 3.0 D.

In Figure 5 the EDoF counterpart IQ Vivity has a similar profile for the periphery beyond *r* = 1.1 mm, but a more complex behavior in its inner 2.2 mm diameter. The innermost region up to *r* = 0.5 mm has a higher power value of almost 21 D for the green wavelength. There is a drop in power below 18 D in the next section reaching up to *r* = 0.65 mm. The curve shape from *r* = 0.65 mm to *r* = 1.1 mm has some resemblance to the combination of fourth- and sixth-order spherical aberrations shown in Figure 1. After starting at approximately 21 D, the profile reaches almost 22 D before decreasing down to 19.5 D and connecting to the outermost zone.

The LuxSmart lens, depicted in Figure 6, has a nearly constant profile for *r*  >  1.1 mm, which indicates no aberrations in this region. The inner part up to 0.9 mm shares a large similarity to the combination of fourth- and sixth-order spherical aberrations with opposite sign in Figure 1. This region starts at approximately 20.6 D for the green wavelength and increases to 21.4 D. As mentioned, Bausch & Lomb designed their lens for this aberration combination in the innermost zone, so the shape matches the expectations. Between the inner and outer zones, the transition is smoothed out, without abrupt changes in refractive power.

As expected, all three lenses show normal dispersion, with refraction being strongest in the blue, followed by green and red. However, the LuxSmart lens has a smaller chromatic power variation compared with the other lenses, which is consistent for a material with a higher Abbe number.

### Ray Propagation

A ray propagation image of the focus zone is suitable to display the beam paths and properties of the depth of focus. Similar to the Power Profile section, the lens is positioned in the vitreous medium with *n_v_* = 1.336 and beams parallel to the optical axis are impinging. But in contrast to a ring source, a circular disk is used here. Depending on the desired pupil state, the disk diameter is either set to *P* = 3.0 mm (photopic) or *P* = 4.5 mm (mesopic). Each of the 50,000 rays is displayed with only a low opacity, so the resulting view is similar to an intensity image of the beam path. The lens power and relative vergences are calculated according to ISO 11979-2,^9^ with the same method as described in the Power Profile section.

**Figure 7.**
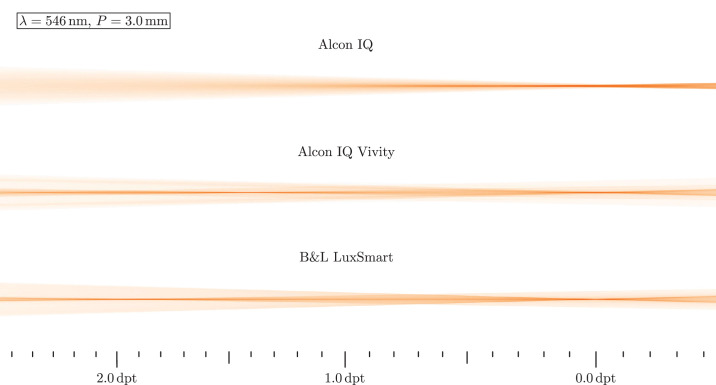
Ray propagation visualization for a monochromatic green source and photopic conditions. B&L, Bausch & Lomb.

**Figure 8.**
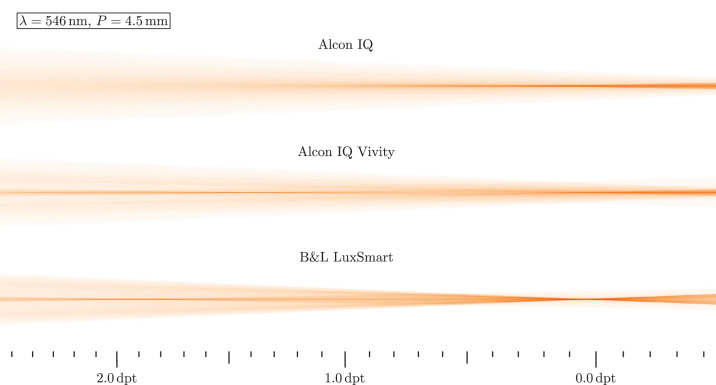
Ray propagation visualization for a monochromatic green source and mesopic conditions. B&L, Bausch & Lomb.

In a first analysis, monochromatic light with wavelength λ = 546 nm hits the lens. The highest axial focus position is interpreted as far focus position, which is set to 0.0 D. From this, relative vergences are calculated and displayed as scale inside the figure. Light travels from left to right in these images. Figure 7 shows the resulting beam paths for daylight and Figure 8 for low light situations.

Under photopic conditions, the Alcon IQ shows a clear and sharp distance focus, while for increasing vergences the rays spread out quickly. Besides the far focus, the IQ Vivity and LuxSmart have a secondary focal region inside the intermediate vision. Its position is at around 1.4 D for the IQ Vivity and around 1.9 D for the LuxSmart. In both cases it can be seen that the rays from inside the lens diameter travel from to the intermediate focus, while the outer rays move to the far focus. The ray density near the optical axis between both foci decreases, however the LuxSmart shows a slightly visible core throughout this region. In the case of an enlarged pupil in mesopic conditions, the lateral beam diameter increases in all cases. The IQ still shows a sharp focus, but now there are more rays outside the focal core. This finding is similar with the IQ Vivity, but here the intermediate focus remains quite sharp. With the LuxSmart, both focus positions are clearly visible and there is not such a large spread at the far focus as with the other two lenses. For both EDoF lenses it is noticeable that more light is now focused toward 0.0 D when compared with the smaller pupil.

**Figure 9.**
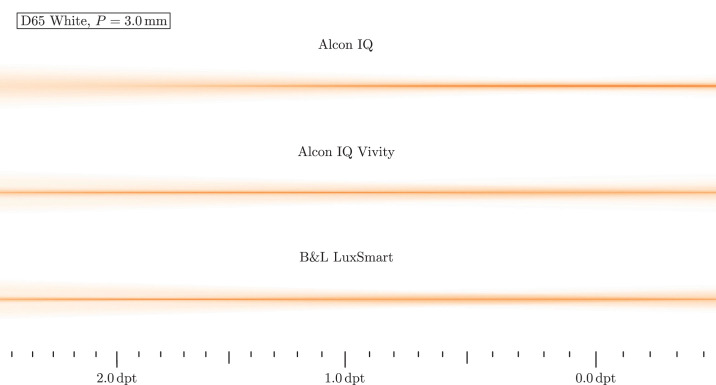
Ray propagation visualization for the D65 white spectrum and photopic conditions. B&L, Bausch & Lomb.

**Figure 10.**
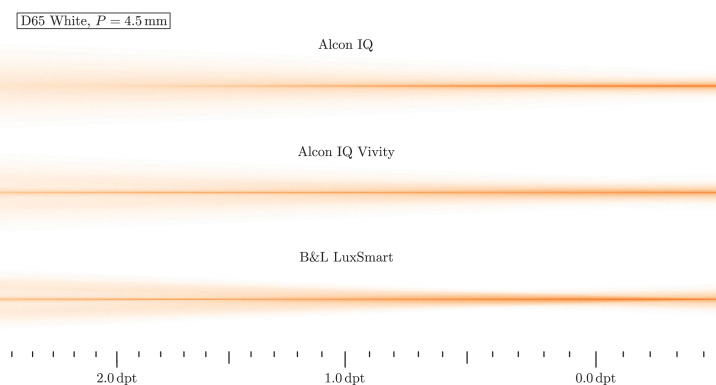
Ray propagation visualization for the D65 white spectrum and mesopic conditions. B&L, Bausch & Lomb.

Nevertheless, monochromatic light does not reflect typical everyday situations. To depict realistic conditions, broadband white light is used in the following. The D65 standard illuminant is appropriate for this task, because it represents average daylight. Each beam is assigned a random wavelength in the visible range of the spectrum, so that all beams in combination reproduce the original spectrum. The resulting beam paths are depicted in Figures 9 and 10.

Figure 9 shows a smaller pupil corresponding to a daylight situation. The Alcon IQ has a longer focal range, visible as a longer focal core, compared with the monochromatic case. With both the Vivity and LuxSmart, the original focal positions are no longer recognizable; due to the dispersion they have become a cylindrical focus that is pronounced over a wide distance. In both cases, it is wider than that of the monofocal Alcon IQ, while reaching well into the region of 1.5 to 2.0 D. When compared with the monochromatic images, all lenses’ beam margins become blurred and the ray intensity no longer ends abruptly, but is continuously decreasing in off-axis direction.

The beam diameters of all lenses increase for mesopic vision shown in Figure 10*.* Analog to the smaller pupil in daylight, the focus range increases with the monofocal lens when compared with monochromatic light. With the two EDoF lenses, a long depth of focus range is created. Compared with to the smaller pupil under photopic conditions, however, there are more rays in the range of 0.0 to 0.5 D, whereas previously they were distributed more evenly. In the far focus, the LuxSmart has a greater intensity of rays around the sharp focus core, which would then manifest itself as glare. With the Vivity, the rays are spread further, but the intensity is significantly reduced. This should therefore result in better image quality. In contrast, the focus core of the LuxSmart is sharper in the 1.0 to 2.0 D range.

Interestingly, one mechanism that increases the depth of focus is longitudinal chromatic aberration. The variation in focal lengths turns distinct monochromatic focal positions to a long focus core. Although the longitudinal chromatic aberration in the power profiles in the Power Profile section seems severe, the ray propagation plots in Figures 7 to 10 do not indicate a significant deterioration in image quality, since the beam diameter is at a comparable scale and a sharp focal region is retained. However, the impact of longitudinal chromatic aberration on visual quality should be subject to further investigation.

The manufacturer-specified spherical aberration is also visible in all beam path images. Negative spherical aberration should be noticeable in the beam paths, because the lenses are not yet integrated into any eye model, so the positive spherical aberration of the cornea does not counteract the negative one. With positive spherical aberration, the paraxial rays have a larger focal length than the peripheral rays, whereas with negative, it is the other way around. A longer focal length for the peripheral rays, indicating such negative spherical aberration, is visible for the IQ and IQ Vivity in all beam path figures. The LuxSmart, however, has its focus clearly pronounced, so it must be aberration free. In this sense, the shown performance of the IQ and IQ Vivity is worse than expected in reality: spherical aberration mainly affects peripheral rays, which for the lenses under investigation run to the far focus. Therefore, far focus sharpness is expected to be improved in a real eye.

## Discussion

### Added Value of EDoF

Our previous results clearly suggest the positive effect of EDoF lenses. In Figures 9 and 10 a distinct extended depth of field in the beam path is seen. Unfortunately, this form of visualization does not allow us to estimate the exact range of sharp vision, but based on the ray path, we estimate a range from 0.0 D into the region of 1.0 to 2.0 D. Near the far focus, the optical performance should be a little worsened, according to the intensities in the beam plots. However, for larger vergences the situation has improved a lot, as there is a sharp focus core and a low intensity beam periphery. It is noteworthy that in all cases the outer beam region is comparable in size between monofocal and EDoF models, so only little additional photic phenomena are expected. Therefore, the Vivity and LuxSmart lenses not only provide significantly improved intermediate vision, but also have comparable disturbing effects to the monofocal model.

These results are in accordance with clinical studies. McCabe et al.^44^ report better immediate vision for the IQ Vivity compared with the IQ, although the latter has slightly better acuity for distance vision. Spectacle independence has increased to 21.6% compared with 3.1% for the IQ lens. Both lenses show comparable photic phenomena. Gundersen and Potvin^45^ and Bala et al.^46^ confirm good intermediate and distance vision and low reports of halo, glares and starburst. For the LuxSmart, similar outcomes are presented in clinical studies. Campos et al.^47^ show similar results for distance vision, but superior intermediate and near acuity when compared with a reference monofocal IOL. Dysphotopsias are low and comparable between both lenses. Improved intermediate vision, comparable distance acuity and low dysphotopsia are confirmed by Tahmaz et al.^48^ and Gawęcki et al.^49^

### Comparison of EDoF Models

The Bausch & Lomb LuxSmart and Alcon IQ Vivity differ in various aspects, such as surface geometry, material, EDoF mechanism and refractive power profile. Due to all these different properties, differences in lens behavior are also expected. But for the smaller pupil beam path in Figure 9 the beam dimensions and intensities actually perform similarly. The only noticeable differences lie in the regions 0.0 to 1.0 D and 1.5 to 2.0 D, where the LuxSmart seems to have a sharper focal core with more intensity.

The larger pupil case in Figure 10 is more interesting. Here, it seems that the Alcon IQ Vivity is designed for greater sharpness for the far focus and the smaller vergences, whereas the Bausch & Lomb LuxSmart rather puts a higher importance on the intermediate focus.

The Bausch & Lomb model thus seems to produce more consistent viewing conditions, regardless of day or night vision and distance, whereas the Alcon lens seems to be optimized for night vision at long distances. In fact, the lenses are marketed to different target groups, the former lens as an all-rounder “for your daily range of vision” and leisure activity,^25^ the other promising “excellent distance, intermediate and functional near vision” for daytime and “clinically proven monofocal visual disturbance profile” for far vision at low light situations.^50^ The IQ Vivity is recommended for frequent night drivers.^51^^,^^52^ Depending on the patient, the more pupil-independent behavior can also be an advantage of the LuxSmart lens.

An overview of results is shown in the Table. It includes values for the through-focus area under the MTF by Azor et al.^53^ to showcase the difference in acuity and range of vision. The range of vision was determined as dioptric distance between the far focus and the intersection of the curve with either 0.0 or 0.2 logarithm of the minimum angle of resolution (logMAR). These visual acuities were estimated from the MTF curves and equation 2 of the aforementioned work. As data for the monofocal Alcon IQ under different pupil states were not available, values for the Johnson & Johnson Tecnis ZCB00 are listed instead. This approach is viable, as Alarcon et al.^54^ describe only minor differences in simulated visual acuity between both lenses. The findings of Azor et al.^53^ confirm better intermediate vision for the LuxSmart over the Vivity regardless of photopic or mesopic conditions, although the Vivity has better distance vision at the larger pupil size.

**Table.** Overview of Results and Mesopic and Photopic Area Modulation Transfer Function (MTF) Data From Azor et al.^53^

	Alcon IQ	Alcon IQ Vivity	Bausch & Lomb LuxSmart
Design	Both surfaces monofocal	Monofocal posterior, EDoF anterior surface	Monofocal posterior, EDoF anterior surface
Spherical Aberration	−0.2 µm	−0.2 µm	None
Power Profile	Negative parabolic	High power center. Surrounding wavefront shaping ring. Periphery negative parabolic	Flat periphery. Inner region with higher power. Smooth transition between both
Pupil dependency	Far vision adapted regardless of pupil state	EDoF (photopic), far vision optimized (mesopic)	EDoF regardless of pupil state
MTF at 0.0, 0.75, 1.5 D^53^	40, 21, 10 (phot)	27, 20, 18 (phot)	27, 23, 18 (phot)
	36, 12, 7 (mes)	29, 15, 10 (mes)	22, 22, 12 (mes)
Range of vision (0.0/0.2 logMAR)^53^	0.7 / 1.7 D (phot)	0.7 / 2.6 D (phot)	1.4 / 2.4 D (phot)
	0.4 / 0.9 D (mes)	0.5 / 1.6 D (mes)	0.8 / 1.9 D (mes)

Data for the similar Tecnis ZCB00 are used for the Area MTF values of the Alcon IQ.

### Comparison With Earlier Work

#### Surface Shape

With regard to the surface shape of the LuxSmart, a similar curve and a comparable zone distribution are found in figures 5 and 10 of a multizonal lens patent.^55^ This patent describes a lens with an inner zone with increased refractive power, as well as a transition zone and an outer zone with decreased curvature and refractive power. The profile of the transition zone from Figure 3 has similarities to figures 4 to 7 in the presumably used patent for the lens.^30^

In the case of the Vivity, the profile has a strongly resembling outline to figures 2 and 4 of the associated patent, where the zone of the X-Wave technology consists of a raised area and three linear sections.^22^ Additionally, the measured shape is consistent with measured data from Tognetto et al.^13^

#### Power Profile

There are several sources for refractive power profiles of IOLs, for instance figure 6 of a Zeiss patent^42^ or figure 6 of the work from Borkenstein and Borkenstein.^41^ These references show basic profile components, which are also present in the curves in our findings in Figures 4, 5, and 6. The Alcon IQ Vivity and IQ both have a negatively parabolic or partially parabolic profile, implying negative spherical aberration, which is consistent with manufacturer claims.^21^ The LuxSmart combines fourth- and sixth-order spherical aberrations of opposite signs in the inner zone and is aberration free in the outermost zone, which matches manufacturer data.^25^

Schmid and Borkenstein^56^ and Borkenstein et al.^57^ characterized Zernike aberrations for both lenses. Unfortunately, it is not possible to directly compare Zernike and Seidel aberrations from the Power Profile section, because they differ in their mathematical form. Nevertheless, both formulations describe the complexity of the wavefront error, where the order of the aberration also indicates the highest polynomial degree. According to both publications, the Vivity has a strongly negative spherical aberration of fourth order and higher terms of sixth and tenth order in the central 2.0 mm diameter part of the lens. This indicates a more complicated refractive power curve. The outer, peripheral region suggests an aberration correcting design.

In the inner part of the LuxSmart fourth- and sixth-order spherical aberrations occur in a similar magnitude with alternating sign, as well as an eighth-order term. The latter arises if parts of the outermost flat zone from Figure 6 are included in the characterization, the shape is thus too complex to be described by only two terms of spherical aberration. The authors also report an aberration-free outer region of the lens.

These results are consistent with our findings and support our conclusions.

#### Ray Propagation

Son et al.^16^ measured the ray propagation for the monofocal Alcon IQ in a physical eye model. Figure 2 of their article depicts the ray paths for pupils of 3.0 mm and 4.5 mm. However, the beam is shown for a larger focal length range than in this work's Figures 7 and 8. A single distinct focal zone and a widening of the lateral extent of the rays for higher and lower vergences is seen, as well as a decrease in intensity. In the enlarged pupil case the rays have a larger lateral extent for all axial locations. These findings are all consistent with our results. The same paper also includes a MTF measurement in figure 2, which shows a decrease of the MTF and a decrease in depth of field for mesopic conditions when compared with a photopic situation.

The work from Baur et al.^19^ shows the light pathways around the focal region for both the Alcon IQ and Alcon IQ Vivity, also measured in a physical eye model. There, figure 2 shows a direct comparison for a pupil of *P* = 3.0 mm, being equivalent to photopic vision. For the Alcon IQ Vivity, two foci are visible, as well as a high intensity region between those two. There is also a lesser intensity peripheral region of the beam. When compared with the monofocal Alcon IQ, the outermost rays have a similar lateral extent. These findings are all in line with our observations. However, a more detailed comparison is not viable, because our lenses are not embedded inside an eye model.

### Limitations

Measurement uncertainties and alignment and positioning errors all cause shape deviations. The choice and quality of the mathematical fitting affect the geometry of the virtual IOL as well. These deviations alter the optical behavior and the refractive power profiles.

For example, it is noticeable that the refractive power in the peripheral region of the LuxSmart lens in Figure 6 is not completely flat, but has small variations around 0.2 D. This is because the refractive power includes a dependence on the radius of curvature, which in turn depends on the second derivative of the surface. Such a twofold differentiation of the processed data, even when smoothed or fitted beforehand, leads to a high sensitivity to errors. Because the modeling is based on height differences, the expected error of the refractive power is largest where the differences are small relative to measurement and processing uncertainties. This is the case in the flat region near the lens center, and for the Vivity in the range *r* = 0.55 mm to *r* = 0.65 mm, where the rising linear segment, visible in Figure 2, cancels parts of the overall lens slope.

Another source of error is an inaccurate refractive index and Abbe number. The available data often specify the index to two decimal places only or do not provide information on the associated wavelength or ambient temperature. This deviation impacts the whole wavefront shape, but is mainly noticeable in a shifted focal position.

Assuming an error for shape and refractive index of 1% to 2%, the resulting refractive power difference is 0.2 to 0.4 D. However, even in the worst case, the relative errors in the relevant investigated properties are small enough that they should not change the meaning and interpretation of results.

Another aspect is that a simulation-based approach using raytracing does not account for wave optical effects. However, as natural scenes consist mostly of incoherent light and the beam diameters in the Ray Propagation section lie above the Rayleigh limit, only small deviations are expected. Additionally, a suitable eye model was not included in our analysis, as the characterization was limited to the behavior and design characteristics of the IOL itself. Clinical acuity, contrast sensitivity and range of vision depend on additional aspects not investigated in this work, including the patient's biometry, the postoperative IOL position and alignment, and neuroadaptation. Hence, the simulation-based approach does not replace in vivo or in vitro studies, but should be seen as supplement to these methods.

## Outlook

With existing lens models and appropriate software, many paths are open for further investigation. For instance, placing a lens in its natural setting using a suitable eye model should lead to even more insights.

An enhancement of the raytracer to include the functionality to render detector images will allow the simulation of natural scenes. For lens analysis, pinhole images or resolution charts are good choices for appropriate benchmarks, as well as a characterization of the point spread function. Furthermore, generating polychromatic images is reasonable, which incorporate dispersion properties and simulate color contrast and impression. An investigation of the impact of tilt, decentration or off-axis incidence could also be easily done in silico.

This analysis should lead to an even better understanding of the mechanisms of action of these state-of-the-art EDoF lenses.
